# Micellar Casein and Whey Powder Hold a TGF-β Activity and Regulate ID Genes In Vitro

**DOI:** 10.3390/molecules26020507

**Published:** 2021-01-19

**Authors:** Layla Panahipour, Selma Husejnovic, Jila Nasirzade, Stephan Semelmayer, Reinhard Gruber

**Affiliations:** 1 Department of Oral Biology, Medical University of Vienna, Sensengasse 2a, 1090 Vienna, Austria; layla.panahipour@meduniwien.ac.at (L.P.); n1350960@students.meduniwien.ac.at (S.H.); jila.nasirzaderajiri@meduniwien.ac.at (J.N.); n01629942@students.meduniwien.ac.at (S.S.); 2 Department of Periodontology, School of Dental Medicine, University of Bern, Freiburgstrasse 7, 3010 Bern, Switzerland; 3 Austrian Cluster for Tissue Regeneration, Donaueschingenstraße 13, 1200 Vienna, Austria

**Keywords:** casein, whey, TGF-β, fibroblasts, epithelial cells, oral health, nutrition

## Abstract

Casein and whey being food supplements have been considered to be used in oral health care products. However, the response of oral cells to micellar casein and whey powder remains unclear. Considering that milk contains the growth factor TGF-β, and lactoperoxidase was recently reported to decrease the expression of inhibitor of DNA-binding (ID) proteins, there is a rationale to assume that casein and whey can also provoke these responses in oral cells. To examine the TGF-β activity, gingival fibroblasts were exposed to reconstituted casein and whey powder from food supplement before the expression of TGF-β target genes were analyzed by reverse transcription-quantitative polymerase chain reaction. Immunoassays were performed for interleukin11 (IL11) in the cell culture supernatant and for TGF-β in the reconstituted casein and whey. We blocked TGF-β by neutralizing the antibody and the TGF-β receptor type I kinase with the inhibitor SB431542. We also showed smad3 phosphorylation and smad2/3 nuclear translocation by Western blot and immunostaining, respectively. Moreover, with reconstituted casein and whey powder, ID1 and ID3 expression analysis was evaluated in HSC2 human oral squamous carcinoma cells. We report here that casein and whey powder caused a robust increase of TGF-β target genes interleukin11 (IL11), NADPH oxidase 4 (NOX4) and proteoglycan4 (PRG4) in gingival fibroblasts that was blocked by SB431542 and the neutralizing antibody. Moreover, casein and whey powder increased the phosphorylation of smad3 and nuclear translocation of smad2/3. No changes of proliferation markers Ki67 and cyclinD1 were observed. Furthermore, reconstituted casein and whey powder decreased ID1 and ID3 expression in the HSC2 oral squamous carcinoma cells. These findings suggest that the processing of milk into casein and whey powder maintains the TGF-β activity and its capacity to regulate ID1 and ID3 genes in oral fibroblasts and oral squamous carcinoma cells, respectively. These data increase the scientific knowledge on the biological activity of casein and whey with a special emphasis on oral health.

## 1. Introduction

Milk, being a hallmark of mammalian evolution, exerts its beneficial effects in newborns undergoing breastfeeding serving as a nutritional supply for milk proteins casein and whey, in addition to carbohydrates, lipids and minerals [1]. Milk should not be restricted to its nutritional aspects being a rich source of the TGF-β [2]. Milk TGF-β is supposed to exert at least part of the beneficial activity of milk in vivo, for example to reduce the allergic reactions in ovalbumin-tolerized mice [3] and to ameliorate tissue damage and mortality in colitis and endotoxemia murine models [4]. In vitro, TGF-β activity is identified by its ability to regulate target genes, most notably interleukin11 (IL11), NADPH oxidase 4 (NOX4) and proteoglycan4 (PRG4) based on independent screening approaches [5,6,7], and some of TGF-β effects in vivo are mediated via IL11 [8,9] and NOX4 [10]. This in vitro bioassay was recently used to confirm that milk [11] but also regular infant formula has a potent TGF-β activity [12]. Nevertheless, casein and whey powder have not been tested for their TGF-β activity. Among the bioactive proteins in milk is also lactoperoxidase with its application in food, cosmetics and medical industries as it exhibits an antimicrobial activity [13]. Lactoperoxidase provokes cellular responses, for example to decrease the expression of DNA-binding protein inhibitor 1 (ID1) and ID3 in HSC2 human oral squamous carcinoma cells [14]. IDs proteins are helix–loop–helix transcription factors regulating cell-cycle progression and cell differentiation [15], also during wound healing [16]. Apart from lactoperoxidase, TGF-β1 can downregulate ID genes in keratinocytes [16]. We want to take advantage of our established bioassays to refine knowledge on how casein and whey may support tissue homeostasis in the oral cavity.

Casein being the major protein in milk [17] and whey remaining as a byproduct in cheese production [18], are processed into powder. The most obvious market for trading micellar casein and whey powder is for athletes seeking to support muscle growth by weight training [19]. Apart from athletes, it is the elderly or even geriatric person who can benefit from milk-based supplements to counteract sarcopenia [20,21]. Preschool children might benefit from milk-based supplements by improved overall nutritional status and psychomotor learning [22]. Even though the consumption of micellar casein and whey powder steadily increase, particularly in athletes and elderly people, the TGF-β activity in oral fibroblasts and the effect of changing ID1 and ID3 in HSC2 oral squamous carcinoma cells has not been reported so far. Finding answers to this question seems relevant as TGF-β [23] as well as lactoperoxidase [24] are rather heat stable molecules.

Casein and whey, apart from serving as food supplements, have received attention in oral health research. For example, the adsorption of bovine milk caseins on the tooth surface was proposed having a positive impact on the prevention of dental diseases by affecting the adhesion of early bacterial colonizers [25,26,27] and reducing demineralization of hydroxyapatite [28,29]. The possible role of TGF-β activity to contribute to oral health, however, remains at the hypothetic level that is based on findings of the gastrointestinal system [3,4]. It is particularly the mixing of toothpaste with lactoperoxidase that has entered the market of health care products [13]. Thus, there is a demand to better understand the influence of casein and whey on oral cells. Considering that regular infant formula holds a potent TGF-β activity [12] that reduces allergic reactions in ovalbumin-tolerized mice [3], it is likely that this is also true for the main ingredient’s casein and whey protein. If, however, casein and whey, similar to milk [14], activate cells of the epithelial cell lineage remains to be studied. The aim of the present study was therefore to investigate casein and whey powder with respect to provoking a TGF-β response in gingival fibroblasts and decreasing ID1 and ID3 expression in HSC2 oral squamous carcinoma cells.

## 2. Results

### 2.1. Casein and Whey Powders Do Not Affect Viability and Proliferation of Gingival Fibroblasts

**Figure 1:**
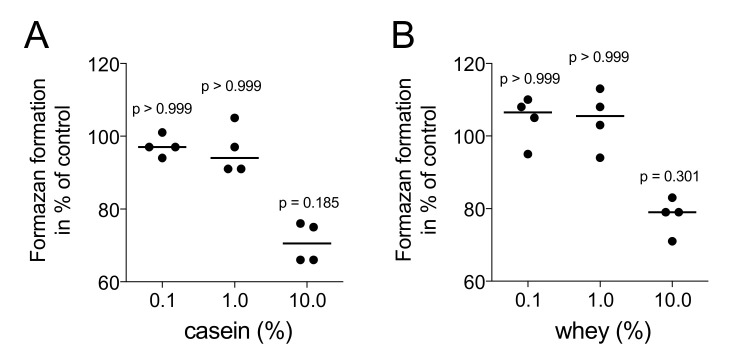
Casein and whey powder at 1% do not affect the viability of gingival fibroblasts. Gingival fibroblasts were incubated for 24 h with 0.1% to 10% (**A**) casein and (**B**) whey. Substrate conversion into solubilized formazan crystals was determined on a photometer. Graphs showing the formation of formazan crystals being expressed as percentage of unstimulated controls (100%). Statistic was based on a Friedmann test.

**Figure 2:**
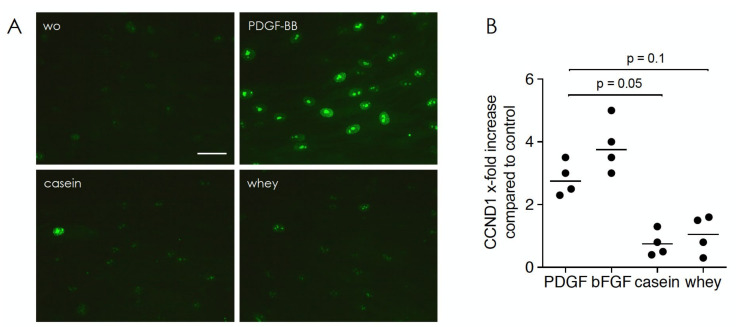
Casein and whey powder at 1% have no impact on cell proliferation. Gingival fibroblasts were incubated for 24 h with 1% casein and whey powder, as well as with 100 ng/mL of the mitogens PDGF-BB and 50 ng/mL bFGF. (**A**) Immunostaining for Ki-67 protein (also known as MKI67), a cellular marker for proliferation and (**B**) the expression of the cell cycle regulator cyclin D1 (CCND1) were determined. Data were normalized for the untreated control being “one”. Statistic was based on a Friedmann test. Scale bar indicates 100 µm. “wo” stands for without, indicating the unstimulated cells.

In a first step, the viability of gingival fibroblasts being exposed to aqueous fraction of casein and whey powder was determined. Formazan formation, indicating the presence of NAD(P)H-dependent cellular oxidoreductase enzymes, was maintained at 1% but decreased at 10% casein (*p* = 0.019) and whey (*p* = 0.301), respectively (Figure 1). We therefore performed the downstream experiments with 1% aqueous fraction of casein and whey powder. Under these conditions, the potent mitogen PDGF-BB but not 1% aqueous fraction of casein and whey powder caused a strong Ki67 nuclear signal and an increased expression of the cell cycle regulator cyclin D1 in gingival fibroblasts (Figure 2).

### 2.2. Casein and Whey Powder Stimulate TGF-β Target Genes Expression in Gingival Fibroblasts

**Figure 3:**
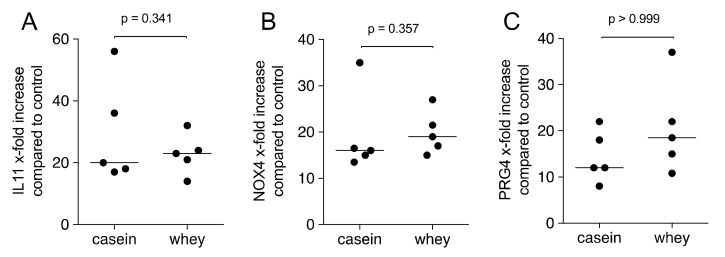
Casein and whey powder enhance TGF-β target genes in gingival fibroblasts. Gingival fibroblasts were exposed to 1% of a pooled reconstituted powder from three providers of casein and whey for 24 h followed by expression analysis of (**A**) IL11, (**B**) NOX4 and (**C**) PRG4. Data points represent fold change of independent experiments compared to the unstimulated controls. Statistical analysis was based on Mann–Whitney U test.

First, the amount of TGF-β1 in the aqueous fraction of 1% casein and whey powder was measured by immunoassay showing median levels of 755.4 pg/mL (min 570.0, max 930.0) and 77.4 pg/mL (min 74.0, max 100.0), respectively. In aqueous fraction of total cow milk, median levels of TGF-β1 were 532.0 pg/mL (min 309.0, max 1161.1). Next, gingival fibroblasts were exposed to the aqueous fraction of casein and whey powder. With 1% casein and whey powder, transcript levels of IL11, NOX4 and PRG4 were increased in gingival fibroblasts using GAPDH for normalization (Figure 3). More insights are provided by showing that the increase of TGF-β target gene is independent of the manufacturer of the casein and whey powder (data not shown).

### 2.3. A TGF-β Neutralizing Antibody Reduce the Whey-Stimulated Gene Expression

**Figure 4:**
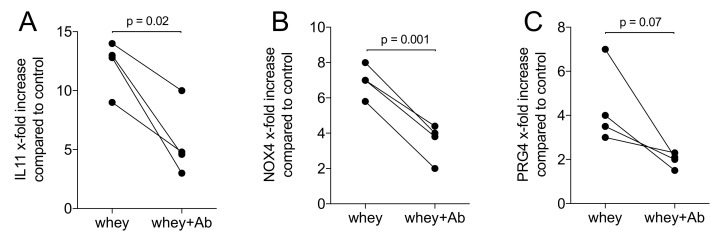
TGF-β pan specific neutralizing antibody reduces whey TGF-β activity. Gingival fibroblasts were exposed to 1% whey powder with and without 10 ng/mL of a TGF-β pan specific neutralizing antibody for 24 h followed by expression analysis of (**A**) IL11, (**B**) NOX4 and (**C**) PRG4. Data points represent fold change of independent experiments compared to the unstimulated controls. Statistical analysis was based on paired T-test.

We then exposed gingival fibroblasts with the aqueous fraction of whey powder in the presence and absence of a TGF-β pan specific neutralizing antibody. When used at 10 ng/mL, the antibody significantly lowered the response of the fibroblasts to aqueous fraction of whey powder (Figure 4). When using casein, the antibody was not effective, likely because of the known blocking activity of casein (data not shown).

### 2.4. Gene Expression Is Suppressed by TGF-β Receptor I Kinase Inhibitor SB431542

**Figure 5:**
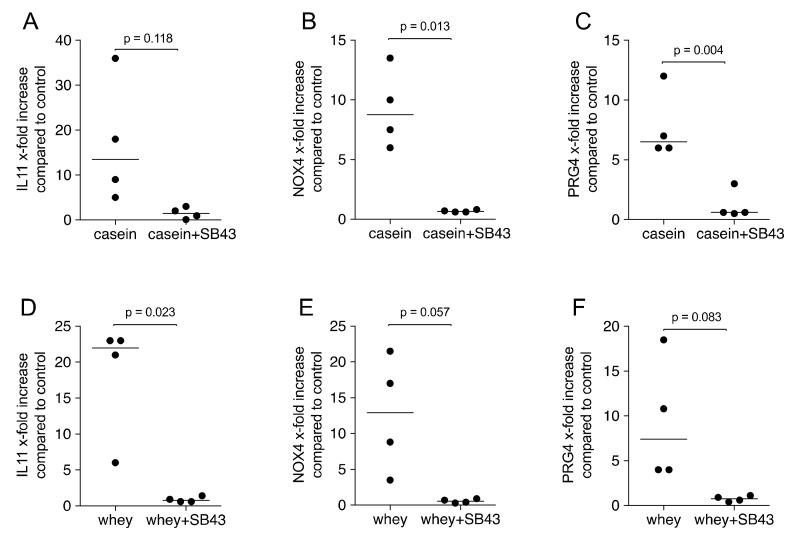
Casein and whey powder formulation enhances TGF-β target genes via TGF-β RI kinase. Gingival fibroblasts were exposed to 1% of a reconstituted pooled casein (**A**–**C**) and whey (**D**–**F**) powder with and without the TGF-β RI kinase inhibitor SB431542 (SB43). Expression analysis of (**A**,**D**) IL11, (**B**,**E**) NOX4 and (**C**,**F**) PRG4 was performed with RT-PCR. Data points represent fold change of independent experiments compared to the unstimulated controls. Statistical analysis was based on paired T-test.

**Figure 6:**
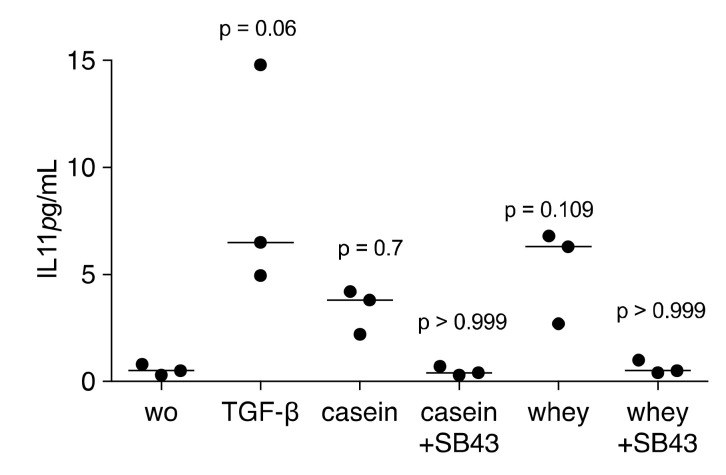
IL11 immunoassay of fibroblasts exposed to cell supernatants. Immunoassay for IL11 was performed with the supernatant of the cells and the data are expressed as pg/mL. TGF-β RI kinase inhibitor SB431542 is marked as SB43. Statistical analysis was based on a Friedman test and Dunn’s multiple comparison. “wo” stands for without, indicating the unstimulated cells.

We further determined that the TGF-β receptor I kinase inhibitor SB431542 can neutralize the effects of casein and whey powder. Pharmacological inhibition of TGF-β receptor I kinase blocked the response of gingival fibroblasts to processed casein and whey powder based on the expression of IL11, NOX4 and PRG4 (Figure 5). In agreement with the findings based on the transcription, also IL11 protein release into the supernatant was prevented by SB431542 (Figure 6). Moreover, IL11 protein levels in the respective supernatant were increased, again at a similar magnitude between casein and whey (Figure 6).

### 2.5. Casein and Whey Stimulate Phosphorylation and Nuclear Translocation of Smad Proteins

**Figure 7:**
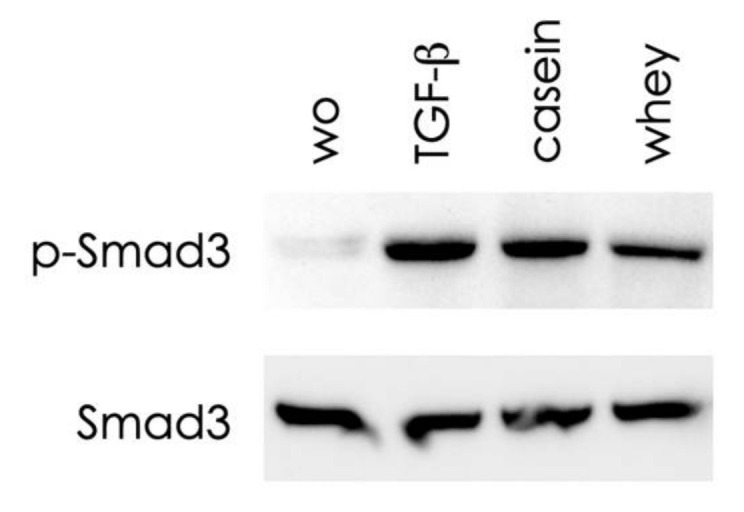
Casein and whey powder enhance phosphorylation of smad3. Serum-starved gingival fibroblasts were exposed to 1% of a reconstituted pooled casein and whey powder for 30 min before being subjected to Western blot analysis of phosphorylation of smad3. Cells exposed to recombinant TGF-β and casein and whey powder caused a strong increase in the phosphorylation of smad3. “wo” stands for without, indicating the unstimulated cells.

**Figure 8:**
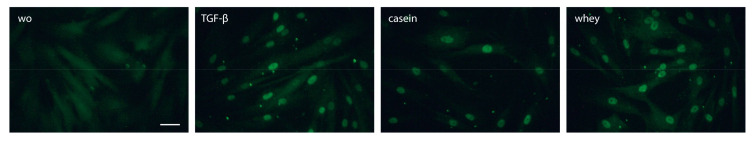
Casein and whey powder enhance smad2/3 nuclear translocation. Serum-starved gingival fibroblasts were exposed to TGF-β (10 ng/mL) and 1% of a casein and whey powder for 30 min before fluorescent labelling of smad2/3. The nuclear signal is visible with cells exposed to recombinant TGF-β and casein and whey powder. Scale bar indicates 100 µm. “wo” stands for without, indicating the unstimulated cells.

To further confirm the activation of canonical TGF-β receptor signaling, we focused on smad2 and smad3 signaling. In support of the activation of the TGF-β receptor, casein and whey caused the phosphorylation of smad3, as identified by Western blot analysis (Figure 7). Considering that activated smads translocate into the nucleus, we report here that casein and whey caused smad2/3 nuclear translocation in gingival fibroblasts (Figure 8), without any obvious differences in the signaling intensity when comparing casein and whey.

### 2.6. Gene Expression Analysis of HSC2 Exposed to Casein and Whey Powder

**Figure 9:**
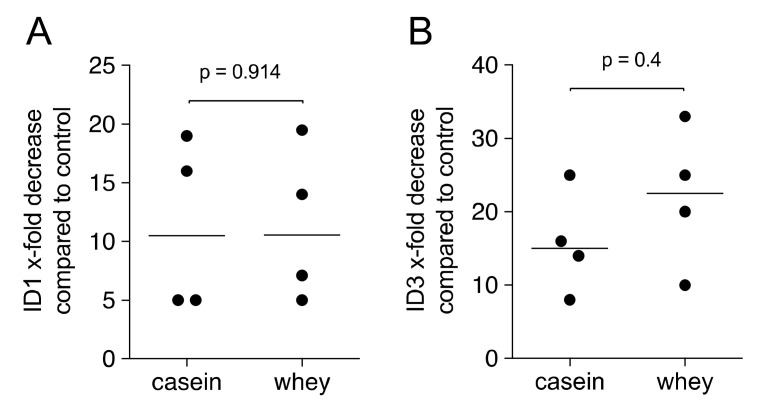
ID1 and ID3 expression in HSC2 exposed to casein and whey powder. HSC2 oral squamous cell carcinoma cells were exposed to 1% casein and whey powder for 24 h, before expression analysis of the target genes (**A**) ID1 and (**B**) ID3 were performed. Data indicate the x-fold decrease compared to unstimulated control cells. Statistical analysis was based on Mann–Whitney U test.

To further prove that cells of the oral epithelial cell lineage are responsive, HSC2 cells were exposed to 1% casein and whey powder. Similar to our recent findings obtained with aqueous fractions of pasteurized milk [6], transcript levels of ID1 and ID3 were decreased with GAPDH used for normalization (Figure 9).

## 3. Material and Methods

### 3.1. Aqueous Fractions of Casein and Whey Powder

Three different batches of casein ((i) ESN Micellar Casein, Fitmart Gmbh & Co. Kg, Elmshorn, Germany; (ii) Casein Zero, BioTechUSA, Szada, Hungary; (iii) Sportnahrung Casein, Sporternährung Mitteregger GmbH, Graz)), and whey powder ((i) ESN Isowhey Hardcore, Fitmart Gmbh and Co. Kg, Elmshorn, Germany; (ii) Iso Whey Zero, BioTechUSA, Szada, Hungary; (iii) Sportnahrung Whey, Sporternährung Mitteregger GmbH, Graz)) were reconstituted with serum-free Dulbecco’s Modified Eagle Medium (DMEM) to reach a 2% solution, always prepared fresh for each experiment. Sportnahrung Casein and Whey have been supplemented with papain and bromelain by the manufacturer. To obtain the aqueous fractions, suspended casein and whey were immediately centrifuged at 10,000× g for 5 min at room temperature. The aqueous fractions were then 0.2 µm filtered (VWR international, Radnor, PA, USA) and either equivolumetricaly pooled or representing the different batches. Prior to cell stimulation, the aqueous fractions were further diluted with DMEM representing a 1% solution of casein and whey.

### 3.2. Primary Gingival Fibroblasts and Oral Squamous Cells

Human gingival fibroblasts were prepared from explant cultures of three independent donors after approval of the Ethical Committee of the Medical University of Vienna (EK Nr. 631/2007). The oral squamous cell carcinoma cell line HSC2, originally obtained from Health Science Research Resources Bank (Sennan, Japan), was kindly provided by Prof. Rausch-Fan, Department of Periodontology, Medical University of Vienna, Austria. Gingival fibroblasts and HSC2 cells were plated in growth medium at 30,000 and 50,000 cells/cm^2^ into culture dishes, respectively. The following day, cells were exposed to the aqueous fractions representing a 1% solution of casein and whey, if not otherwise indicated. Gingival fibroblasts were also exposed to 10 ng/mL recombinant human TGF-β1 (ProSpec-Tany TechnoGene Ltd., Rehovot, Israel) in serum-free medium for 24 h, before gene expression analysis was performed. SB431542, a TGF-β receptor I kinase inhibitor, was used at 10 µM (Calbiochem, Merck Millipore, Darmstadt, Germany). The TGF-β neutralizing pan-specific polyclonal rabbit IgG AB-100-NA (R&D Systems, Minneapolis, MN, USA) was used at 10 ng/mL. Platelet-derived growth factor (PDGF-BB) (R&D Systems, Inc., Minneapolis, MN, USA) was used at 100 ng/mL; and basic fibroblast growth factor (bFGF, FGF2) (50 ng/mL; Strathmann Biotech AG, Hamburg, Germany) was used in indicated experiments. Cell culture supernatant was harvested, centrifuged and stored frozen until subjected to immunoassay. Fibroblasts expanded for less than 10 passages were used for the experiments.

### 3.3. Viability Assay

For viability experiments, gingival fibroblasts were incubated with aqueous fractions of casein and whey at the indicated concentrations. After 24 h, an MTT (3-[4,5-dimethythiazol-2-yl]-2,5-diphenyltetrazolium bromide; Sigma, St. Louis, MO, USA) solution at a final concentration of 0.5 mg/mL was added to each well of a microtiter plate (CytoOne, Starlab International, Hamburg, Germany) for 2 h at 37 °C. Medium was removed and formazan crystals were solubilized with dimethyl sulfoxide. Optical density was normalized to unstimulated control values.

### 3.4. qRT-PCR Analysis and Immunoassay

Total RNA was isolated with the ExtractMe total RNA kit (Blirt S.A., Gdańsk, Poland). Reverse transcription was performed with SensiFAST^TM^ cDNA (Bioline, London, UK). Polymerase chain reaction was done with the SensiFAST™ SYBR ROX Kit (Bioline, Luckenwalde, Germany) on a CFX Connect™ Real-Time PCR Detection System (Bio-Rad Laboratories, Hercules, CA, USA). Primer sequences are hPRG4_F CAGTTGCAGGTGGCATCTC, hPRG4_R TCGTGATTCAGCAAGTTTCATC; hNOX4a_F TCTTGGCTTACCTCCGAGGA, hNOX4a_R CTCCTGGTTCTCCTGCTTGG; hGAPDH_F AAGCCACATCGCTCAGACAC, hGAPDH_R GCCCAATACGACCAAATCC, hID1_F CCAGAACCGCAAGGTGAG, hID1_R GGTCCCTGATGTAGTCGATGA; hID3_F CATCTCCAACGACAAAAGGAG, hID3_R CTTCCGGCAGGAGAGGTT. hCCND1_F TCGGTGTCCTACTTCAAATGTGT; hCCND1_R GGGATGGTCTCCTTCATCTTAG. The IL11 primer was from Bio-Rad (qHsaCEP0049951). The mRNA levels were calculated by normalizing to the housekeeping gene GAPDH using the ΔΔCt method. The amount of TGF-β1 in the aqueous fraction of 1% casein and 1% whey powder was measured by TGF-β1 Quantikine ELISA (#DY240; R&D Systems, Minneapolis, MN, USA). For the IL11 immunoassay, gingival fibroblasts were exposed to 1% of a reconstituted pooled casein and whey powder. After 24 h, the cell culture supernatant was harvested and subjected to the human IL11 Quantikine ELISA testing (#DY218; R&D Systems). ELISA data were not normalized to an internal compound.

### 3.5. Western Blot Analysis

Gingival fibroblasts were serum-starved for 24 h and then preincubated for 30 min with 1% casein and whey powder. Cell extracts containing SDS buffer and protease inhibitors (PhosSTOP with cOmplete; Sigma, St. Louis, MO, USA) were separated by SDS-PAGE and transferred onto nitrocellulose membranes (Whatman, GE Healthcare, General Electric Company, Fairfield, CT, USA). Membranes were blocked and the binding of the first antibody raised against p-smad3 (rabbit; phospho S423 + S425; EP823Y, Abcam, Cambridge, UK) and the smad3 (mouse; Smad3 (38-Q): sc-101154, Santa Cruz Biotechnology, SCBT, Santa Cruz, CA, USA) were detected with the appropriate secondary antibody linked to a peroxidase. Chemiluminescence signals were visualized with the ChemiDoc imaging system (Bio-Rad Laboratories, Inc., Hercules, CA, USA).

### 3.6. Immunofluorescence

Gingival fibroblasts exposed to reconstituted casein and whey powder for 24 h were incubated with anti-smad2/3 antibody (D7G7 XP^®^ Rabbit mAb, Cell Signaling, Danvers, MA, USA) and with Ki67 (8D5, Mouse mAb antibody, 9449 Cell Signaling Danvers, MA, USA) for 24 h at 4 °C. Following blocking by 1% BSA and permeabilization with 0.1% Triton X, an Alexa Fluor^®^ 488-conjugated secondary antibody (Cell Signaling) was added for 1 h at room temperature. Images were captured under a fluorescent microscope (Axio Imager M2, Carl Zeiss AG, Oberkochen, Germany).

### 3.7. Statistical Analysis

All experiments were repeated at least three times. Data from individual experiments are shown as dot-blots. Statistical analysis was based on Friedmann test (Figure 1, Figure 2 and Figure 6), Mann–Whitney U test (Figure 3 and Figure 9) and paired T-test (Figure 4 and Figure 5). Data were analyzed by the Prism 8.0e software (GraphPad Software; San Diego, CA, USA). The *p*-values are indicated in the respective figures.

## 4. Discussion

This study assumes that micellar casein and whey powder contributes to the biological activity of food supplements and possibly have beneficial effects on oral health. In support of this assumption, we show here that aqueous fraction of micellar casein and whey powder are rich in TGF-β activity as indicated by the increased expression of the respective target genes IL11, NOX4 and PRG4 involving the canonical TGF-β receptor I kinase and smad3 signaling pathway in gingival fibroblasts. The TGF-β activity is not related to changes of the proliferation marker protein Ki-67 and cyclinD1 expression in gingival fibroblasts. Moreover, we provide data that aqueous fraction of micellar casein and whey powder exert the same activity as pasteurized milk in the oral squamous epithelial cell line HSC2 by decreasing the ID1 and ID3 genes [14]. The latter is an indirect evidence for the presence of lactoperoxidase, a heat stable milk enzyme, in casein and whey powder. We thus show that casein and whey maintain the basic in vitro properties of pasteurized milk we have recently reported [11,12,14].

Our data thus confirm the TGF-β activity of milk and dairy products including whey [11] and infant formula [12] based on the activation of TGF-β receptor I kinase expression of IL11 and NOX4 in oral fibroblasts, both genes that mediate the fibrotic activity of TGF-β in cardiovascular and liver fibrosis [8,9] and acute kidney injury and pulmonary fibrosis [10,30], respectively. PRG4 is involved in mediating TGF-β in osteoarthritis in mice [31]. These findings should not be extrapolated to a possible fibrotic activity of micellar casein and whey powder but to support the use of the target genes in a bioassay. Moreover, our observation that the TGF-β pan specific neutralizing antibody blocked TGF-β activity of whey but not casein can be explained by the unspecific cross-reactivity between the casein and antibodies. The TGF-β activity has no effect on fibroblast proliferating as micellar casein and whey powder failed to change Ki-67 and cyclinD1, while PDGF-BB and bFGF, both strong mitogens, greatly increased staining and expression, respectively. This finding is in line with the rather low and biphasic mitogenic activity of TGF-β in periodontal fibroblasts [32]. Whey proteins even prevent the expression of Ki-67 in UV irradiated cells [33]. PDGF-BB and bFGF are strong mitogens for mesenchymal cells [34], thus in line with the increased Ki-67 staining [34] and cyclinD1 expression [35]. Also, in line with our previous data is that aqueous fraction of micellar casein and whey powder decrease the ID1 and ID3 genes in HSC2 cells presumably via lactoperoxidase activity [14]. The present study is thus another piece of evidence that processing of milk into casein and whey powder maintains the TGF-β and presumably also the lactoperoxidase activity.

TGF-β and seemingly also the lactoperoxidase activity survives the processing of milk into the final products. Casein is precipitated by acidification of skim milk, cooked and washed. The acid casein is then neutralized and spray-dried to obtain caseinates and milled to produce casein powder. Whey was originally considered as the soluble serum proteins that remain after precipitation of casein during cheese production. Other methods to gain whey proteins are based on ion exchangers [36] and membrane filtration [37]. TGF-β is heat stable and resists low pH, they are both conditions that can even increase the activity of the preforms of the growth factor [23]. Similarly, lactoperoxidase is among the minor whey proteins that are heat stable and also resist pH changes. Lactoperoxidase is resistant in vitro to acid pH 3 and to human gastric juice [38]. Moreover, the heat stability of lactoperoxidase is used as an index of pasteurization efficiency in milk [24]. Obviously, the manufacturers of casein and whey proteins included do not provide insights into the manufacturing process, thus no conclusions on the impact of the processing of milk into casein and whey on TGF-β and possibly also lactoperoxidase activity can be drawn. The TGF-β activity of Sportnahrung Casein and Whey being supplemented with papain and bromelain by the manufacturer can be explained by the fresh preparation of the aqueous fraction and immediate cell exposure. 

The clinical relevance of the finding that micellar casein and whey powder, independent of the manufacturer, strongly increase TGF-β target genes and decrease ID1 and ID3 genes, remains unclear but leaves room for speculations. Considering that milk TGF-β can reduce the allergic reactions in ovalbumin-tolerized mice [3] and ameliorate tissue damage in colitis and endotoxemia murine models [4], some beneficial effects of the TGF-β in micellar casein and whey powder can also be assumed. Particularly in athletes, micellar casein and whey powder support muscle growth upon weight training [19]. Maybe it is also TGF-β that exerts some beneficial effects in the gastrointestinal system in the elderly person taking milk-based supplements [20,21] and even systemic effects of dietary TGF-β should not be ruled out. Support for this assumption comes from preclinical research showing that the oral administration of TGF-β1 can protect against gastrointestinal diseases and lower systemic IL6 and IFN-γ levels based on a necrotizing enterocolitis model [39] and summarized in a review on maternal TGF-β and immunological outcomes [40]. Thus, future research should identify if the in vitro TGF-β activity of micellar casein and whey powder can be translated into a clinically relevant beneficial effect that presumably exceeds the oral health system.

Not so easy to translate is the clinical meaning of decreased ID1 and ID3 genes in oral squamous oral epithelial cell line HSC2 [14]. ID proteins are overexpressed and ID1 may serve as an independent prognostic factor to predict survival time of human oral squamous cell carcinomas [41,42] but also in other squamous cell carcinoma entities [43]. It can be speculated that micellar casein and whey powder might exert some beneficial effect by returning the IDs to normal levels. In addition, ID1 and ID3 are usually known as target genes for BMP4, but in this case ID1 and ID3 are increasingly expressed [44]. Also, TGF-β increased the expression of ID1 and ID3 in prostate cancer cell lines [45] and so far, only lactoperoxidase was reported to decrease ID1 and ID3 expression on oral squamous oral epithelial cell line [14]. Interestingly, ID1 and ID3 can downregulate extracellular components induced by TGF-β, including fibronectin and collagen in various cell types [44]. Apart from the lack of knowledge on the clinical meaning of decreased ID1 and ID3, it remains open if it is lactoperoxidase in micellar casein and whey [46] being responsible or not for the gene expression changes. Considering that toothpaste and mouth rinses with lactoperoxidase are available under the trade names Biotène^®^ Dry Mouth Moisturizing Spray; Zendium^TM^, Orabarrier^TM^, Bioxtra^®^ as summarized recently [13], we might suggest studies, first to determine the amount of lactoperoxidase and then using micellar casein and whey powder as supplements for oral health care products.

In summary, our data provide convincing evidence that micellar casein and whey powder are a rich source of TGF-β based on expression changes of the respective target genes in oral fibroblasts, while the support for the lactoperoxidase activity being responsible for the decreased ID1 and ID3 in oral squamous epithelial cells is indirect. This research might serve as a scientific basis to further investigate the effects of micellar casein and whey powder that go beyond their nutritional aspects.

## Data Availability

All raw data are made available on request.
